# Probiotics: Evolving as a Potential Therapeutic Option against Acetaminophen-Induced Hepatotoxicity

**DOI:** 10.3390/biomedicines10071498

**Published:** 2022-06-24

**Authors:** Saikat Dewanjee, Tarun K. Dua, Paramita Paul, Abhijit Dey, Jayalakshmi Vallamkondu, Sonalinandini Samanta, Ramesh Kandimalla, Vincenzo De Feo

**Affiliations:** 1 Advanced Pharmacognosy Research Laboratory, Department of Pharmaceutical Technology, Jadavpur University, Kolkata 700032, India; 2 Department of Pharmaceutical Technology, University of North Bengal, Raja Rammohunpur 734013, India; tarunkduaju@gmail.com (T.K.D.); paramita37@gmail.com (P.P.); 3 Department of Life Sciences, Presidency University, Kolkata 700073, India; abhijit.dbs@presiuniv.ac.in; 4 Department of Physics, National Institute of Technology, Warangal 506004, India; vlakshmij@gmail.com; 5 Department of Dermatology (Skin & Venereology), Employee’s State Insurance Corporation Medical College & Hospital, Patna 801103, India; snspresi95@gmail.com; 6 Department of Biochemistry, Kakatiya Medical College, Warangal 506007, India; ramesh.kandimalla@gmail.com; 7 Applied Biology, Council of Scientific and Industrial Research-Indian Institute of Chemical Technology, Tarnaka 500007, India; 8 Department of Pharmacy, University of Salerno, 84084 Fisciano, Italy

**Keywords:** acetaminophen, hepatotoxicity, nutraceuticals, postbiotics, probiotics

## Abstract

Acetaminophen (APAP) is the most common prescription medicine around the world for the treatment of pain and fever and is considered to be a safe drug at its therapeutic dose. However, a single overdose or frequent use of APAP can cause severe acute liver injury. APAP hepatotoxicity is a prevalent cause of acute liver disease around the world and the lack of suitable treatment makes it a serious problem. In recent years, there has been a surge in interest in using probiotics and probiotic-derived products, known as postbiotics, as health and disease negotiators. A growing body of evidence revealed that they can be equally effective against APAP hepatotoxicity. Different probiotic bacteria were found to be pre-clinically effective against APAP hepatotoxicity. Different postbiotics have also shown exciting results in preclinical models of APAP hepatotoxicity. This review summarized the protective roles and mechanisms of the different probiotic bacteria and postbiotics against APAP hepatotoxicity, with critical discussion. A brief discussion on potential novel probiotics and postbiotics for oxidative liver injury was also included. This review was written in an attempt to pique the interest of researchers in developing a safe therapeutic option against oxidative liver damage using probiotics and/or postbiotics as dietary supplements.

## 1. Introduction

Acetaminophen (APAP), commonly known as paracetamol, is the most routinely prescribed antipyretic and analgesic agent for all age groups under the class of non-steroidal anti-inflammatory medicine drugs (NSAID) [1]. Over 100 over-the-counter analgesics and antipyretics contain APAP [2]. Pediatricians routinely prescribe it to treat fevers in children. APAP is included in several prescription medications as a single-drug formulation or in combination with other drugs for effective and safe management of pain and fever [1]. However, a single overdose or frequent application of APAP has been revealed to develop acute hepatotoxicity, and even death, due to acute hepatocellular injury [3]. APAP-induced hepatotoxicity first appeared in the United States in the mid-1980s, and since then APAP-induced hepatotoxicity has frequently appeared [4,5]. APAP induces mild to severe hepatotoxic effects ranging from hepatic transaminase elevations, cholestasis, or hepatitis to acute liver failure [5]. The clinicopathological features of APAP-induced hepatotoxicity include hepatic redox insult, cirrhosis, fibrosis, inflammation, apoptosis, and even malignancies [5,6,7]. A small portion of hepatic APAP undergoes phase I oxidation by the action of cytochrome P450 enzymes (CYPs), mainly CYP 2E1, to generate N-acetyl p-benzoquinone imine (NAPQI), a toxic intermediate. NAPQI builds up in the liver during an acute overdose or frequent application of APAP, which can directly bind to cellular proteins, especially to the mitochondrial membrane proteins resulting in loss of membrane integrity [8]. In addition, NAPQI can also bind to reduced glutathione (GSH), resulting in a suppression of hepatic GSH level, which consequently causes augmented oxidative stress to the liver via enhanced accumulation of oxidative and nitrosative free radicals [9,10]. Oxidative stress coupled with mitochondrial dysfunctions leads to hepatocellular damage [8,10]. Though APAP-induced acute hepatotoxicity emerges as a critical challenge, so far N-acetylcysteine is the single clinically approved antidote for APAP hepatotoxicity that exists on the market. N-acetylcysteine boosts endogenous redox defense by activating glutathione (GSH). However, N-acetylcysteine exhibits side effects and is not effective in cases of severe APAP overdoses [11,12]. Some agents, such as 4-methyl pyrazole (CYP blocker), and calmangafodipir (superoxide dismutase mimetic) have shown realistic prospects against APAP hepatotoxicity in humans [12]. Despite this, scientists and clinicians are still looking forward to an effective therapeutic drug to counteract APAP poisoning. Probiotics and postbiotics have emerged as potential nutraceuticals with promises to improve the quality of life and prevent diseases [13]. Probiotics and postbiotics have also shown promise to rescue the liver from diseases and xenobiotic-induced hepatotoxicity, and restore normal liver functions [14,15,16,17,18,19,20,21,22].

Probiotics are nonpathogenic viable microorganisms, including certain commensal bacteria that confer health-promoting and disease-preventing attributes to the host when administered in a proper dose. The salient features of a probiotic strain include being adequately characterized, remaining viable throughout its shelf-life in a formulation, and having at least one successful human trial as evidence of its efficacy and safety [23]. Probiotics mainly consist of lactic acid bacteria, including many strains of Lactobacillus, Bifidobacterium, Streptococcus, and Enterococcus. Among them, Lactobacillus and Bifidobacterium are available in different fermented milk products and also are commercially available in the market as nutraceuticals or functional food [24,25]. Gut microorganisms play a significant role in maintaining liver health [15]. Thus, modulation of gut microbiota represents a potential approach to hepatoprotection. In this aspect, probiotics may play a therapeutic role against liver injury via restoring gut microbiota. A growing body of evidence revealed the prophylactic roles of probiotics against liver damage by restoring the gut microbial population to strengthen the integrity of the intestinal wall, reducing bacterial translocation and epithelial invasion, and mitigating endotoxemia [14,16]. They can simultaneously activate the production of antimicrobial peptides and stimulate host immunity [14,26]. In addition, probiotics can attenuate oxidative and inflammatory liver damage [14,15]. Several preclinical studies revealed that probiotics can prevent oxidative stress-induced acute liver injury [17,18,19,20]. This review summarizes the protective effects of probiotics against APAP-induced liver injury. However, the trends are aiming at replacing live bacteria with non-viable bacterial components or metabolites to minimize the risk associated with the supplementation of live microorganisms [27,28]. Postbiotics are metabolic byproducts or non-viable probiotic components in the host that exhibit biological effects on the host, either directly or indirectly. The non-viable probiotic components include bacterial cell lysates, extracellular polysaccharides, peptidoglycan-derived muropeptides, functional proteins, and pili-type structures [29]. The metabolic by-products of probiotics are typically produced by the probiotic bacteria that utilize prebiotics, which include dietary supplements or foodstuffs comprising non-digestible components supporting the growth and proliferation of probiotic bacteria. Postbiotics could be similarly effective against APAP hepatotoxicity because they exert pleiotropic effects on many cellular processes [21,22]. In this review, we also briefly addressed the therapeutic potentials of postbiotics against APAP hepatotoxicity.

## 2. APAP Hepatotoxicity: A Critical Health Issue

**Figure 1:**
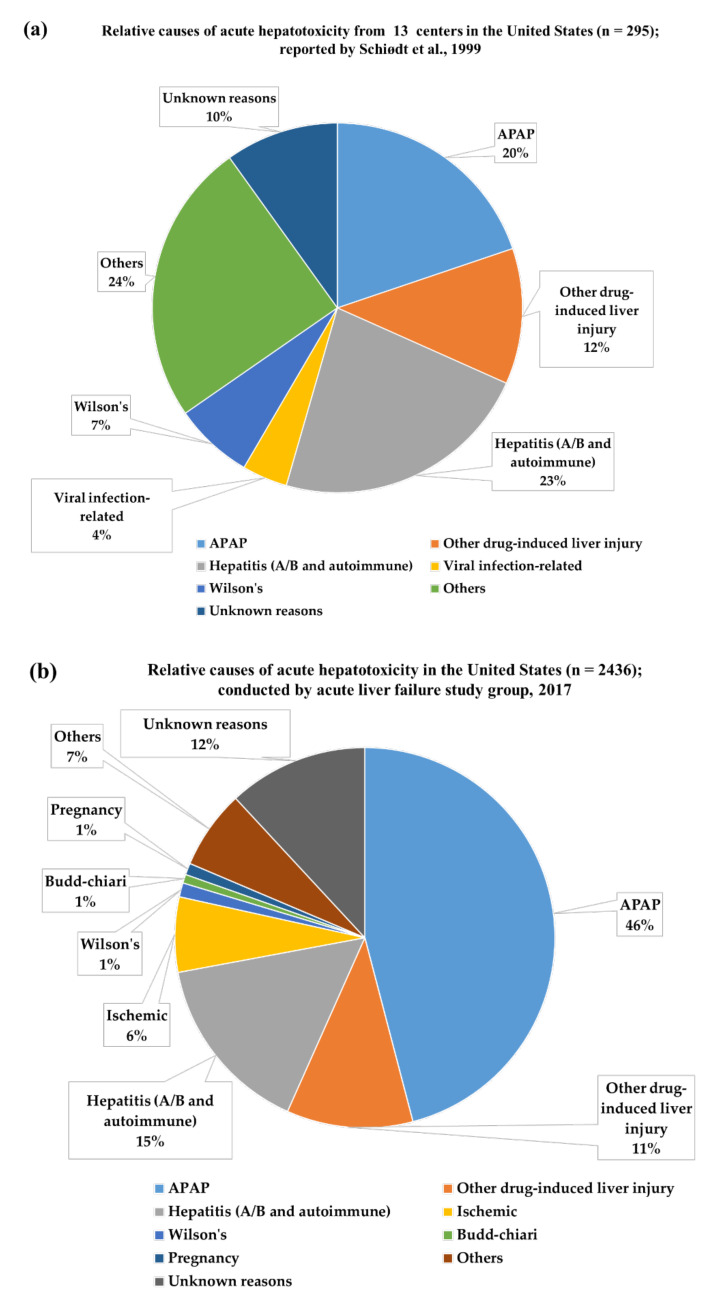
Relative frequency of acute hepatotoxicity caused by different factors in the United States. (**a**) In the United States, between 1994 and 1996, there was a 20% incidence (*n* = 295) of acute APAP poisoning leading to acute liver failure [34]. (**b**) The incidence of acute APAP hepatotoxicity was increased to 42% (*n* = 2436) in 2017 [36].

Due to the widespread availability and accessibility of APAP, APAP hepatotoxicity is a prevalent means of self-poisoning around the world. It represents a high risk of mortality with a 50% death risk if left untreated [30]. Generally, most APAP hepatotoxicity results from ‘unintentional overdose’ or ‘therapeutic misadventure’ or regular intake of APAP without consulting a medical practitioner. APAP hepatotoxicity has been widely reported in the United Kingdom, United States, Canada, Portugal, Germany, France, and Australia [31,32]. Acute liver failure (ALF) caused by an APAP overdose is most common (60–75% of ALP etiology) in the United Kingdom [31]. Every year, around 30,000 people are admitted to hospitals in the United States for treatment of APAP hepatotoxicity [5]. APAP hepatotoxicity accounts for approximately half of all cases of ALP and is the foremost reason for liver transplantation in the United States [33]. A retrospective study found a 20% incidence of acute APAP toxicity (Figure 1a) leading to ALF in the United States from 1994 to 1996 [34]. In the United States, the rate of APAP hepatotoxicity grew by 42% between 1998 and 2003, and it continues to rise [35]. According to a survey of the ALP study group in 2017, APAP-poisoning has caused 46% of 2436 cases of acute hepatotoxicity in the United States (Figure 1b) and 40–70% of all cases in the United Kingdom and Europe over the last 40 years [36]. In a report, it has been claimed that APAP hepatotoxicity is more prevalent in women as compared to men in the United States [37]. According to recent reports, hospitalizations for intentional APAP overdose increased by 108% in Australia from 2004 to 2017, and the trend is continuing [38,39]. Similarly, the prevalence of acute APAP toxicity, due to unintentional overdosing, is not uncommon [11]. In contrast, APAP hepatotoxicity is less common in Asia, or only represents mild hepatotoxicity in Asia, as compared to western countries [31,40]. A review of 1024 cases of APAP overdose, showed that Asians are more tolerant of APAP overdose [31].

## 3. Mechanistic Insight of APAP Hepatotoxicity

**Figure 2:**
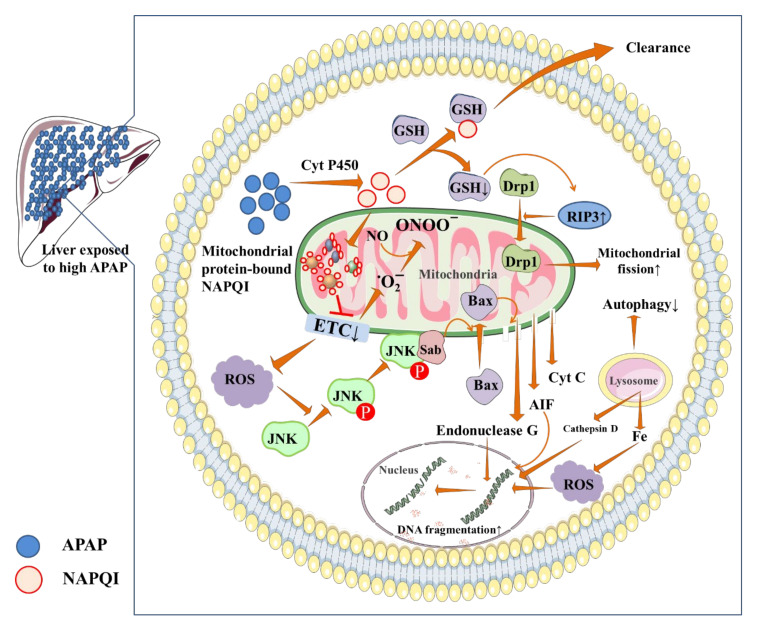
Mechanism of APAP-induced acute liver injury. In an acute overdose or frequent application of APAP, a greater amount of APAP is metabolized by CYP2E1 resulting in an enhanced NAPQI accumulation in the liver, which enhances the accumulation of ROS by depleting GSH and inhibiting the mitochondrial electron transport chain. ROS react with NO to produce RNS which, in collaboration with ROS, impart oxidative damage to liver cells. ROS also activate several pathological signal transductions. In addition, NAPQI-mediated release of lysosomal proteases potentiates hepatocellular necrosis and suppresses autophagy. Lysosomal iron release to the cytosol further triggers ROS production. Brown arrows represent downstream events. ‘↑’ represents upregulation and ‘↓’ represents downregulation. AIF: apoptosis-inducing factor, APAP: acetaminophen, Bax: Bcl-2 associated X, CYP2E1: cytochrome P450 2E1, Cyt C: cytochrome C, Drp1: dynamin-related protein 1, ETC: electron transport chain, GSH: reduced glutathione, JNK: c-jun N-terminal kinase, NAPQI: N-acetyl p-benzoquinone imine, NO: nitric oxide, RIP3: receptor-interacting protein kinase 3.

The mechanism of APAP hepatotoxicity is complicated, particularly concerning the nature of liver cell death. Over the years, several reports have mentioned the nature of cell death is necrosis which mainly occurs in APAP hepatotoxicity. In contrast, since the cells simultaneously demonstrated multiple unique features of apoptosis, some reports implied that the nature of cell death is apoptosis. However, it is well accepted that NAPQI, a highly toxic intermediate that is generated during APAP metabolism, is the principal cause of APAP hepatotoxicity. At the therapeutic doses, around 85–90% of APAP is metabolized to harmless molecules by UDP-glucuronosyltransferase and sulfotransferase and subsequently eliminated through the urine [10]. Little APAP is excreted unchanged; while the remaining 5–9% of APAP undergoes oxidative metabolism by the action of CYPs, mainly CYP 2E1 to a highly toxic intermediate, NAPQI [10]. NAPQI is eliminated in physiological conditions via conjugation with GSH [41]. In acute overdose or frequent application of APAP, glucuronidation and sulfation of APAP are overwhelmed and a greater amount of APAP is metabolized by CYP2E1, resulting in an enhanced NAPQI accumulation in the liver, which immediately depletes hepatic GSH, resulting in an enhanced accumulation of reactive oxygen species (ROS) in the liver [10,41]. In addition, NAPQI binds to proteins containing sulfhydryl groups, especially mitochondrial proteins, and inhibits the mitochondrial electron transport chain (ETC) resulting in a leakage of electrons [10]. Thus, NAPQI endorses superoxide radical production in mitochondria, which subsequently reacts with nitric oxide to form peroxynitrite, a reactive nitrogen species (RNS) that imparts nitrosative stress. GSH generally detoxifies ROS and RNS, thus GSH depletion caused by NAPQI potentiates the accumulation of these free radicals [41]. An increase in oxidative stress enhances mitochondrial membrane permeability resulting in a decrease in mitochondrial membrane potential and hampers ATP production [42]. In addition, NAPQI can directly interact with the α-subunit of ATP-synthase resulting in an impairment of ATP synthesis [43]. Mitogen-activated protein kinase (MAPK) is a class of redox-sensitive signal proteins, which are involved in hepatocellular injury during APAP toxicity. Apoptosis signal-regulating kinase1 (ASK1) is one of the first MAPKs, which has been implicated in APAP hepatotoxicity. ASK1 endorses sustained C-jun-N-terminal kinase (JNK) activation via phosphorylation. Phosphorylated-JNK undergoes translocation to the mitochondria and binds to Sab located on the mitochondrial outer membrane, which further endorses the production of superoxide from ETC. JNK also triggers the translocation of Bax into mitochondria, which further decreases the integrity of the mitochondrial membrane and, as a consequence, endonuclease G, cytochrome C, and apoptosis-inducing factor (AIF) are released into the cytosol [42,44,45]. AIF and endonuclease G can contribute to DNA fragmentation after translocating to the nucleus [42,44,45]. In addition, RIP3 activation is noted in APAP-induced hepatotoxicity, which endorses activation and translocation of dynamin-related protein 1 (Drp1) to mitochondria resulting in mitochondrial fission and mitophagy induction [46,47]. Drp1 translocation to mitochondria is thought to be a downstream effect of JNK activation, which is inhibited by Sab suppression [48]. In addition to mitochondria, lysosomes play a key role in APAP-induced liver damage by releasing lysosomal proteases and iron into the cytosol, which can induce hepatic injury [41]. Iron directly triggers ROS production. The release of cathepsin D, a lysosomal protease to cytosol induces lysosome-mediated necrosis [49]. Furthermore, because the lysosome is a major regulator of autophagy, lysosomal instability leads to autophagy dysfunction. APAP-induced hepatotoxicity has been linked to abnormal autophagy and mitophagy, which causes hepatocyte damage [50,51]. The mechanism of APAP hepatotoxicity has been depicted in Figure 2.

Focusing on the nature of cell death in APAP hepatotoxicity, despite some markers of apoptosis sharing the characteristic expression of induction of apoptosis, the cell death in APAP carries more features of necrosis. Several studies reported the suppression of anti-apoptotic proteins and activation of caspases in APAP hepatotoxicity [44,46,52,53]. Despite some findings claiming that Bcl-2 transcription is reduced in APAP hepatotoxicity, it is unclear whether Bcl-2 is involved in the cell death mechanism or just produces a secondary consequence of the damage repairing. Secondly, the lack of protective effect of pan-caspase inhibitors in APAP-induced liver damage suggests that there is no involvement of caspase-dependent apoptosis in APAP hepatotoxicity. In addition, the lack of apoptotic characteristics in cell morphology and the fall of cellular ATP supports the absence of apoptosis in APAP hepatotoxicity. Since, the cell death shares some basic features of apoptosis, such as Bax translocation to mitochondria, increase in mitochondrial membrane permeability, decrease in mitochondrial membrane potential, the release of cytochrome C, and Smac (second mitochondria-derived activator of caspase) to the cytosol, it is not justified to categorize it as true necrosis. Thus, it would be worthy to describe the nature of cell death as programmed-necrosis.

## 4. Hepatoprotective Mechanisms of Probiotics

**Figure 3:**
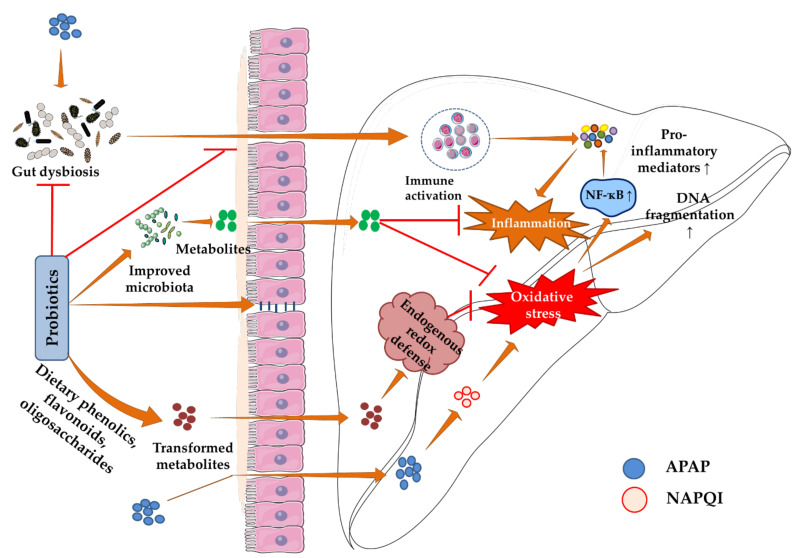
Protective mechanism of probiotics against APAP hepatotoxicity. Probiotics act by altering the composition and activity of the gut flora, resulting in the production of good metabolites that directly reduce APAP-induced oxidative stress and inflammation. Probiotics also attenuate gut dysbiosis, restore intestinal homeostasis, and gut barrier integrity, as well as prevent bacterial translocation to the liver that causes immune hyperactivation. In addition, they produce transformed metabolites (postbiotics) using dietary phenolics, flavonoids, and oligosaccharides, which can activate Nrf-2 and other antioxidant genes that suppress oxidative stress. Brown arrows represent downstream events. Red lines represent inhibition and ‘↑’ represents upregulation. APAP: acetaminophen, CYP2E1: cytochrome P450 2E1, NF-κB: nuclear factor kappa-light-chain-enhancer of activated B cells, Nrf-2: nuclear factor erythroid 2–related factor 2.

A growing body of evidence has revealed the therapeutic roles of probiotics in various liver diseases, including hepatic encephalopathy, fatty liver diseases (alcoholic and nonalcoholic), cirrhosis, sclerosing cholangitis, hepatocellular carcinoma, etc. [15,54,55]. Probiotics reduce oxidative stress, inflammation, and fibrosis, all of which play a role in the etiology of a variety of liver diseases [15,56,57]. Probiotics primarily work by altering the composition and activity of the normal gut flora. Maintenance of a healthy microbiome in the intestine restores intestinal homeostasis that improves ATP production and prevents negative metabolic effects on the liver. Organic acids that are produced by probiotic bacteria can infiltrate the cell membrane, inhibiting nutrient transport and ATPase activity [58]. In addition, probiotics improve gut barrier integrity, preventing hepatic translocation of pathogenic bacteria. These pathogenic bacteria can directly harm the liver by releasing endotoxins and other toxic components [15]. Translocation of pathogenic bacteria to the liver causes immune hyperactivation which triggers hepatic inflammatory cascade via MAPKs (p38, JNK, etc.), interferon regulatory factor 3 (IRF3), and nuclear factor kappa-light-chain-enhancer of activated B cells (NF-κB) activation, resulting in hepatic inflammation. Thus, probiotics prevent hepatic necro-inflammation by restoring gut barrier integrity [58]. In addition, several reports showed that probiotics can suppress hepatic oxidative stress which is a common pathological phenomenon in various liver diseases [59,60,61]. Probiotic bacteria have been found to lower oxidative stress through a variety of mechanisms [62,63]. Probiotics can activate the host endogenous antioxidant system and trigger antioxidase activities. Several probiotic bacteria are known to activate nuclear factor erythroid 2-related factor 2 (Nrf-2) signaling in the host, and, as a result, stimulate the production of host antioxidant enzymes and antioxidases [63]. They produce various metabolites with ROS scavenging potential, such as reduced glutathione (GSH), folate, indole-3-propionic acid, and butyrate. Probiotics also restrict ROS generation by interfering with ROS-producing enzymes, such as NADPH oxidases, CYPs, and cyclooxygenases [63]. In addition, the metal chelating effect of probiotic bacteria potentiates their antioxidant capacity [63]. A report showed that probiotics can catabolize non-absorbable dietary phenolics to small molecular weight phenolics which are absorbed through the gut and produce antioxidant effects [64]. Thus, it could be said that probiotics offer hepatoprotection through a variety of mechanisms (Figure 3).

## 5. Emerging Role of the Gut Microbiota in APAP Hepatotoxicity

APAP hepatotoxicity is significantly regulated by the gut microbiome. Intestinal dysbiosis makes people more vulnerable to APAP-induced liver damage. Schneider and co-workers (2021) analyzed a cohort comprising 500,000 participants in the British Biobank and established that intestinal dysbiosis increases the risk of APAP-induced ALF [65]. In an experimental model, Nlrp6^−/−^ mice that represented intestinal dysbiosis exhibited more susceptibility to APAP hepatotoxicity as compared to wild-type mice [66]. Several preclinical studies have validated the contribution of intestinal dysbiosis to APAP hepatotoxicity [66,67,68]. *Clostridium difficile*, a pathogenic bacterium in the intestine, can produce p-cresol which is metabolized in the liver by the action of sulfotransferases. Sulfotransferases are also involved in sulfation-mediated APAP metabolism in the liver to yield non-toxic metabolites. Thus, p-cresol can compete with APAP and increase APAP hepatotoxicity [68]. Moreover, the expressions of CYPs, which are key enzymes involved in hepatic APAP metabolism, have a strong relationship with gut microorganisms. Germ-free mice exhibiting lower expression of CYP-1A2 and CYP-3A4 enzymes are more able to tolerate APAP hepatotoxicity [69]. Some studies showed that germ-free mice exhibited more tolerance toward APAP toxicity than specific pathogen-free or non-germ-free mice; while a report claimed that intestinal microbiota does not cause any significant difference in susceptibility to APAP hepatotoxicity [69,70,71]. However, the effect of intestinal microbiota on APAP hepatotoxicity largely depends on the type of microbes present in the gut. Diversity in gut microbiota can influence susceptibility to APAP hepatotoxicity. The same strain of mice supplied by different vendors can exhibit diversity in gut microbiota and represent differential responses toward APAP over-dosing. Mice with a higher abundance of Mucispirillum, Turicibacter, and Ruminococcus sp. in the gut are more vulnerable to APAP hepatotoxicity. However, the cohousing of mice received from different vendors could abrogate this differential response, due to the mutual transfer of microbiota between the mice [72]. The presence of *L. rhamnosus* GG in the gut of *Drosophila* and mice represented a higher expression of Nrf-2 in the liver compared to germ-free *Drosophila* and mice. Nrf-2 is a transcription factor that improves redox defense by endorsing the transcriptions of antioxidant enzymes, thus exhibiting protection against APAP toxicity [73]. In another report, Zheng and colleagues (2020) showed that a reduction in gram-positive count in the intestine by vancomycin treatment in experimental mice could increase the 2-hydroxybutyric acid level in the cecum and serum, resulting in a decrease in APAP bioavailability and an increase in GSH level to ameliorate APAP hepatotoxicity [74]. Thus, the pharmacokinetics of APAP can be affected by changes in the abundance, and/or diversity, of gut microbiota. Treatment with *L. reuteri* was shown to increase the degradation of APAP to 68% in mice [75]. Therefore, it is quite obvious that the abundance, and/or diversity of gut microbiota has a critical influence on APAP hepatotoxicity. However, the precise regulatory role of the gut microbial population in APAP hepatotoxicity is yet to be revealed. The same strain of animals supplied by different vendors or maintained in different conditions (foods, water, and housing) can show a difference in the composition of the gut microbiome [67]. Thus, a thorough gut microbiome analysis is necessary before executing any experiment to understand the precise role of gut microbiota in APAP hepatotoxicity. A summary table has been included to show how the composition of gut microbiota differentially affects APAP hepatotoxicity (Table 1). It is proven that probiotic bacteria supplementation promotes liver health and lessens xenobiotic-induced hepatotoxicity by restoring a balance between symbiotic and pathogenic bacteria in the gut and suppressing pathological events in the liver. The hepatoprotective roles of probiotics and their metabolites/non-viable derivatives (postbiotics) against APAP hepatotoxicity have been discussed in the subsequent sections.

## 6. Protective Roles of Probiotic Strains against APAP Hepatotoxicity

The hepatoprotective roles of probiotics and their protective mechanism against APAP hepatotoxicity have been revealed in different preclinical studies [78,79,80,81]. In these reports, different probiotic strains exhibited different mechanisms of hepatoprotection against APAP hepatotoxicity. In addition to their inherent mechanisms, such as reconditioning gut microbiota, maintaining a healthy gut barrier, improving ATP production, and preventing negative metabolic effects, probiotics also ensured antioxidant, immunomodulatory, and anti-inflammatory mechanisms to counteract APAP hepatotoxicity. Moreover, probiotics have been shown to regulate various signaling events against APAP hepatotoxicity [73,81]. A few reports showed that a few probiotic strains can interfere with the pharmacokinetics of APAP [75]. In addition, probiotics use prebiotics to produce some metabolic products, such as short-chain fatty acids, that can attenuate APAP hepatotoxicity [81]. A schematic overview of the overall hepatoprotective mechanism of probiotics against APAP hepatotoxicity is depicted in Figure 2. The hepatoprotective roles of individual probiotics against APAP-induced liver injury are discussed hereunder.

### 6.1. Enterococcus lactis IITRHR1

*E. lactis* IITRHR1, a probiotic strain, was isolated from cottage cheese and exhibited good adhesion to intestinal epithelial cells. In an experimental model of APAP hepatotoxicity, pretreatment with *E. lactis* IITRHR1(10^9^ CFU/day) for seven days, followed by the APAP treatment (1 g/kg/day) for 14 days, exhibited a protective effect against APAP-induced liver damage in male Wistar rats [80]. Oral treatment of *E. lactis* IITRHR1 significantly reversed APAP-induced increase of aspartate aminotransferase (AST), alanine aminotransferase (ALT), and alkaline phosphatase (ALP) levels in the sera, which confirms its hepatoprotective role against APAP hepatotoxicity. E. lactis IITRHR1 treatment corrected APAP-provoked hepatic oxidative stress and its associated pathological signal transduction, DNA fragmentation, and cell death in the hepatic tissue of experimental rats. *E. lactis* IITRHR1 ensured protection against oxidative damage of cellular macromolecules, such as lipids, proteins, and nucleic acids, evidenced by increase in lipid peroxidation, protein carbonylation, and DNA fragmentation. This probiotic also caused a significant reduction in caspase 3, caspase 9, Bax, and cytochrome C activation and reversed Bcl-2 suppression in the murine liver. In this study, *E. lactis* IITRHR1 treatment significantly endorsed redox defense by triggering the levels of endogenous mitochondrial antioxidant enzymes, such as SOD, CAT, GST, and GPx, and antioxidant metabolite, GSH in the livers of experimental rats, which has been regarded as the protective mechanism of *E. lactis* IITRHR1 against APAP hepatotoxicity. The overall protective effect of *E. lactis* IITRHR1 was comparable to that of vitamin C, a standard antioxidant used as a positive control in this study [80]. Though it was claimed in this study that *E. lactis* IITRHR1 could attenuate APAP-induced intrinsic apoptotic signaling by promoting redox defense in the murine liver, the lack of data makes it too early to speculate on the exact nature of cell death. We have discussed in the earlier section of this manuscript that cell death shares some basic features of apoptosis, but cell death carries more features of necrosis. It is not warranted to forecast the nature of cell death without flow cytometric data, the use of specific signal protein inhibitors, the investigation of additional pathological signaling implicated in APAP hepatotoxicity, and the measurement of hepatocellular ATP concentration. However, from this experimental outcome, it can be concluded that *E. lactis* IITRHR1 is beneficial against APAP-induced hepatocellular damage, which may be attributed to the antioxidant effect of this probiotic strain.

### 6.2. S. salivarius ssp. thermophilus St.sa

*S. salivarius* ssp. *thermophilus* also known as *S. thermophilus* is a non-pathogenic and anaerobic lactic acid bacterium. It is a homofermentative facultative probiotic strain that has been used in the production of fermented dairy products for a long time [24]. In a preclinical assay, *S. thermophilus* (10^9^ CFU/day) pretreatment for 7 days could significantly attenuate hepatocellular injury in female Wistar rats caused by a single high dose (200 mg/kg, i.e., 2/3rd of LD_50_) of APAP on day 7 [79]. The hepatoprotective effect of *S. thermophilus* was evidenced by the reduction in the levels of liver function markers, such as ALT, AST, and ALP in the sera of experimental rats. *S. thermophilus* treatment significantly reciprocated both the transcriptions and enzymatic activities of SOD and CAT in the liver. *S. thermophilus* treatment also caused an increase in hepatocellular GSH level and decreased APAP-induced lipid peroxidation in rat liver. In addition, *S. thermophilus* significantly exhibited radical scavenging, metal chelating, and H_2_O_2_ neutralizing potential in vitro. Thus, it could be said that the protective effect of *S. thermophilus* against APAP hepatotoxicity was mainly mediated through an antioxidant mechanism to alleviate APAP-induced oxidative liver injury in rats [79]. However, more study is required at the molecular level to understand the exact mechanism of action.

### 6.3. Bacillus Spores

Bacterial spores, usually from the *Bacillus* species, have probiotic attributes with significant antioxidant, anti-inflammatory, and immunomodulatory properties [82]. MegaSporeBioticTM, a *Bacillus* spore blend comprising *B. licheniformis*, *B. indicus*, *B. subtilis*, *B. clausii*, and *B. coagulans* spores is a broad-spectrum probiotic that maintains a healthy gut barrier and reconditions the gut by improving its microbial diversity. This spore blend has been shown to be effective against acute hepatotoxicity caused by APAP overdose in male Charles River Wistar rats [78]. The oral treatment of spore blend (10^9^ CFU/day) for 12 days to experimental rats significantly reciprocated the augmented AST and ALP levels caused by a single high dose of APAP (2 g/kg). In addition, it has been shown to attenuate APAP-induced augmentation in the levels of proinflammatory mediators, namely TNF-α, IL-1β, and ZO-1, and promoted total antioxidant capacity [78]. However, more studies are required to find out the mechanistic pathway of the hepatoprotective role of Bacillus spores against APAP hepatotoxicity.

### 6.4. L. ingluviei ADK10

*L. ingluviei* ADK10 is a lactic acid bacterium that was first isolated from the chicken intestine and possesses beneficial probiotic properties with good tolerance, hydrophobicity, and adherence in the gastrointestinal tract [83]. This probiotic strain exhibited significant radical scavenging, antioxidant, and reducing potentials in vitro [84]. *L. ingluviei* ADK10 was found to attenuate APAP-induced augmented oxidative stress in the liver of male Wistar rats [84]. APAP (500 mg/kg/day, intraperitoneal) treatment for seven days significantly induced hepatic oxidative stress evidenced by enhancement in lipid peroxidation and depletion of endogenous antioxidants, such as SOD, CAT, and GSH in the liver and blood. Seven-day treatment of *L. ingluviei* ADK10 (10^9^ CFU/day) significantly inhibited APAP-induced oxidative stress by reducing lipid peroxidation and improving endogenous antioxidant levels in both liver tissue homogenate and blood. It is worth noting in this study that this probiotic treatment was able to reverse APAP-induced depletion of SOD (blood and liver), CAT (blood), and GSH (liver) levels to levels that were higher than those observed in normal control animals [84].

### 6.5. L. acidophilus LA14

*L. acidophilus* LA14 is a probiotic lactic acid bacterium that attributes several health benefits by enriching beneficial bacteria and depleting opportunistic pathogens in the gut, degrading oxalates, producing bacteriocin, and improving immune response [85]. Moreover, it can alter the distributions of different metabolites, which appears to be advantageous to health [86]. Recently, the hepatoprotective effect of *L. acidophilus* LA14 has been reported in mice with an acute APAP overdose. In this study, mice pretreated with *L. acidophilus* LA14 (6 × 10^8^ CFU/day, orally) for seven days significantly reciprocated the augmentation of AST, cholinesterase, total bile acids, and total bilirubin, and reduction of total proteins in sera caused by a single oral dose of APAP (300 mg/kg). This probiotic also reduced APAP-induced hepatic inflammation evidenced by a reduction in IL-1α levels in sera. In addition, *L. acidophilus* LA14 treatment significantly attenuated APAP-provoked hepatic hemorrhage, nuclear shrinkage in hepatocytes, and infiltration of inflammatory cells as seen in the liver sections. The authors did not substantiate any hepatoprotective mechanism of *L. acidophilus* LA14 against acute APAP hepatotoxicity. However, in the same investigation, the authors attempted to establish the hepatoprotective mechanism of *L. acidophilus* LA14 against D-galactosamine-induced hepatotoxicity in rats. D-galactosamine causes hepatotoxicity by causing oxidative stress via generating excessive free radicals and inflammation in the liver, which resembles the mechanism of APAP hepatotoxicity [87]. *L. acidophilus* LA14 pretreatment was found to reciprocate D-galactosamine-induced activation of focal adhesion, extracellular matrix-receptor interaction, inflammation, and proteoglycans by regulating the transcriptions of key genes involved in these signaling events. This probiotic strain also activated ascorbate/aldarate metabolism, cysteine/methionine metabolism, PPAR signaling, and peroxisome pathways to impart an hepatoprotective effect [86]. From these observations, we can perceive the hepatoprotective mechanism of *L. acidophilus* LA14 against APAP hepatotoxicity.

### 6.6. L. rhamnosus GG

*L. rhamnosus* GG is a probiotic strain of lactic acid bacteria. *L. rhamnosus* GG pretreatment (2 × 10^8^ CFU/day) for 14 days could significantly attenuate hepatotoxicity in mice caused by a single sub-lethal dose of APAP (300 mg/kg) on day 14, as evidenced by a significant reduction of AST level in the sera [73]. It was found that *L. rhamnosus* GG significantly decreased hepatic oxidative stress and hepatic necrosis. In search of the mechanism, it was found that 5-methoxyindoleacetic acid, a metabolic byproduct of *L. rhamnosus* GG could directly activate Nrf-2, which has been confirmed by measuring luciferase activity of different metabolites of the bacterium. As a consequence of the transcriptions of its downstream factors, such as NAD(P)H quinone dehydrogenase 1 (NQO1), heme oxygenase 1 (HO-1), and glutamate-cysteine ligase catalytic subunit (GCLC) becoming enhanced, 5-methoxyindoleacetic acid could endorse the stabilization and nuclear translocation of Nrf-2. Thus, it would be worthy to mention that *L. rhamnosus* GG could ensure hepatoprotection not only through its fundamental biocatalytic and probiotic activities but also through its metabolic byproduct (5-methoxyindoleacetic acid), which induces bioprocessing pathways in the host by activating the transcriptions of Nrf-2 and its downstream antioxidant genes [73].

### 6.7. L. reuteri K8

*L. reuteri* K8 treatment showed a significant reduction in the oral bioavailability of APAP, evidenced by a 68.4% reduction in the area under the curve in APAP-treated mice as compared to normal control mice [75]. It was found to increase APAP degradation itself and increase APAP degradation in the gut by enhancing arylsulfate sulfotransferase activities without affecting intestinal metabolic activity. In search of the mechanism, it was shown that *L. reuteri* K8 treatment could increase its number adherence in the upper portion of the small intestine and directly influence the composition of gut microbiota. *L. reuteri* K8 treatment increased the population of bifidobacteria, clostridia, and enterococci in the gut, which may have a key role in APAP metabolism [75]. Though there is no direct evidence of its protective role against APAP hepatotoxicity, its role in APAP pharmacokinetics may attribute a therapeutic role in acute APAP hepatotoxicity.

### 6.8. Akkermansia muciniphila

*A. muciniphila* is one of the most common intestinal symbionts regarded as a promising probiotic candidate in the future. In a recent report, oral treatment of *A. muciniphila* (3 x 10^9^ CFU/day) for 2 weeks efficiently suppressed hepatotoxicity caused by a sub-lethal dose of APAP (300 mg/kg, i.p.), evidenced by the reduction in ALT and AST levels and hepatocellular necrosis in mice. *A. muciniphila* treatment significantly attenuated APAP-induced oxidative stress by enhancing hepatic GSH and SOD, suppressed production of pro-inflammatory cytokines (IL-1β, IL-2, IL-6, and TNF-α), alleviated infiltration of macrophages and neutrophils, and prevented DNA fragmentation [81]. In regards to the molecular mechanism, *A. muciniphila* caused activation of the PI3K/Akt pathway, suppression of ERK/JNK activation, and reversal of Bax activation and Bcl-2 suppression. In addition, *A. muciniphila* treatment reciprocated APAP-induced disturbances in gut barrier function, gut dysbiosis, and reduction in short-chain fatty acid production [81]. In this report, although the histological sections of APAP-treated mouse liver showed the signs of severe necrosis, in the subsequent section of the manuscript, the authors mentioned that the nature of cell death was apoptosis and substantiated this with TUNEL assay and the expressions of Bax, Bcl-2, phospho-JNK, phospho-ERK, phospho-Akt, and phospho-PI3K. Of note, neither the TUNEL assay nor the expressions of the abovementioned proteins could predict the exact nature of cell death. However, from the experimental observation, it was obvious that *A. muciniphila* could alleviate APAP-induced acute liver injury by regulating gut microbiota and metabolism, suppressing oxidative stress and inflammation, and regulating various signaling events.

### 6.9. Laktera Nature: A Probiotic Formulation

Laktera nature is a Bulgarian probiotic formulation comprising 25 × 10^9^ CFU/g of live and latent *L. Bulgaricus* DWT1, *L. helveticus* DWT2, *L. lactis* DWT3, and *S. thermophilus* DWT4, 5, 6, 7, 8. Treatment of this probiotic formulation (800 and 1600 mg/kg) for 14 days could attenuate acute hepatotoxicity caused by a single overdose of APAP (1200 mg/kg), evidenced by the reduction in ALT, AST, ALP, and γ-glutamyl transferase levels in the sera of male Wistar rats [88]. However, the molecular mechanism of hepatoprotection was not revealed in this report. A summary table (Table 2) has been included to explain the hepatoprotective effect of the aforementioned bacteria at a glance against APAP hepatotoxicity.

In addition to the aforementioned probiotics, several other probiotic bacteria have shown hepatoprotective effects in different preclinical assays against xenobiotic-induced oxidative liver damage with mechanisms resembling that of APAP hepatotoxicity. *C. butyricum*, *L. salivarius* LI01, *Pediococcus pentosaceus* LI05, *L. fermentum*, *L. plantarum*, *Lactiplantibacillus plantarum* 1201, *Lactococcus lactis*, etc. showed protective effects against CCl4-induced oxidative liver damage by endorsing redox defense and suppressing inflammation in the liver [89,90,91,92,93]. *L. plantarum*, *B. infantis*, *L. casei* Zhang, *L. helveticus* R0052, *B. longum* R0175, *L. reuteri*, etc. were shown to attenuate D-galactosamine-induced oxidative liver damage [94,95,96,97,98]. *Akkermansia muciniphila*, *P. pentosaceus*, *L. paracasei* GKS6, *L. plantarum* GKM3, *L. rhamnosus* GKLC1, *L. plantarum* HFY09, etc. exhibited potential hepatoprotective role against alcohol-induced oxidative liver damage [99,100,101,102]. Given their protective mechanisms against xenobiotic-induced liver damage, the aforementioned probiotics can also be targeted to investigate their potential protective effect against APAP hepatotoxicity.

## 7. Role of Postbiotics against Liver Diseases

Postbiotics are either microbial components or soluble biologically active molecules which are generally produced by probiotics by using prebiotics (non-digestible ingredients of functional foods). Postbiotics include peptides, peptidoglycan-derived muropeptides, bacterial polysaccharides, bacterial surface proteins, short-chain fatty acids, eichoic acids, enzymes, organic acids, etc. [103]. Due to the fact that their usage is safer, compared to living microorganisms, and their having defined chemical identity, long shelf-life, and pleiotropic health-promoting attributes, postbiotics are becoming more and more popular as nutraceuticals [103,104]. These postbiotics can exert both local (in the gut) as well as systemic (to the liver, adipose tissue, circulatory system, etc.) actions. Their local actions include immunomodulatory, anti-inflammatory, and antimicrobial effects; while systemic effects include antioxidant, hypolipidemic, antihypertensive, antiproliferative effects, etc. [103]. Several postbiotics have exhibited potential hepatoprotective roles in preclinical studies. Gut bacteria, such as *C. sporogenes*, *Peptostreptococcus anaerobius*, etc., can catabolize dietary tryptophan to various indole metabolites which are capable of preventing liver disease manifestations by several mechanisms, including aryl hydrocarbon receptor activation, mucus promotion, barrier function improvement, tight junction restoration, glucagon-like peptide-1 activation, antioxidant, and anti-inflammatory attributes [105,106,107]. Gao and colleagues identified a novel secreted protein which exhibited a potential protective effect against lipopolysaccharide/D-galactosamine-induced liver injury [108]. Considering the role of hepatic oxidative stress and inflammation in the pathogenesis of APAP-induced liver damage, the aforementioned postbiotics could equally be effective against APAP toxicity. Recently, the hepatoprotective effect of bacterial short-chain fatty acids, such as acetic acid, butyric acid, and 2-methyl butyric acid, against APAP hepatotoxicity has been mentioned [81]. Some postbiotics have been revealed to show a hepatoprotective role against APAP hepatotoxicity, as discussed below.

### 7.1. 4-Phenylbutyric Acid (PBA)

PBA is a short-chain terminal aromatic substituted fatty acid that is produced naturally during fermentation by colonic bacteria [109]. It is a clinically approved drug for the treatment of familial cholestasis type 2, urea cycle disorders, thalassemia, sickle cell disease, spinal muscular atrophy, and neurodegenerative diseases [110]. Shimizu and colleagues [111] studied the effects of the sodium salt of PBA (Na-PBA) on acute hepatotoxicity caused by APAP overdose in mice. It was shown that pretreatment with Na-PBA (100 and 200 mg/kg) 1 h before APAP (400 mg/kg, i.p.) treatment could significantly reverse APAP hepatotoxicity, evidenced by a significant reduction in the levels of serum ALT, blood ammonia, nitrotyrosine formation, hepatocellular centrilobular necrosis, and DNA fragmentation. It also reciprocated APAP-induced activation of Xbp1 splicing and JNK phosphorylation. Na-PBA pretreatment did not cause any significant change in GSH level, CYP2E1 expression, and NAPQI level in the liver. Post-treatment with Na-PBA administered at 1 or 2 h after APAP treatment also significantly attenuated hepatotoxicity, evidenced by changes in blood parameters, hepatocellular necrosis, and DNA fragmentation. In contrast to Na-PBA pretreatment, post-treatment did not cause any significant change in X-box binding protein-1 (Xbp1) splicing and JNK phosphorylation. The exact protective mechanism of Na-PBA was not deciphered in this study; although the authors claimed that the protective effect may be somehow correlated to that of suppression of DNA fragmentation in hepatocytes [111]. In another study, post-treatment with Na-PBA (120 mg/kg, i.p.) 4 doses at an interval of 3 h starting at 0.5 h after APAP (450 mg/kg, i.p.) treatment up to 12 h significantly attenuated APAP hepatotoxicity [112]. Na-PBA significantly inhibited APAP-provoked activation of activating transcription factor 6 (ATF6) and phosphorylated-JNK protein levels, as well as reciprocated APAP-mediated increase in binding immunoglobulin protein (BiP), and spliced Xbp1, and C/EBP homologous protein (CHOP) genes. In addition, PBA treatment prevented Bax activation and oxidative stress, and hepatocellular necrosis. Similar to that of the previous study, Na-PBA treatment did not show any effect on APAP-induced GSH depletion. Although in the first study, Na-PBA post-treatment did not exhibit any effects on Xbp1 splicing and JNK phosphorylation in the liver, it prominently regulated spliced Xbp1 mRNA and JNK phosphorylation in the second study, which might have been achieved by the multiple dosing of Na-PBA in the second study. From both the studies, it may be predicted that the protective mechanism of Na-PBA against APAP hepatotoxicity is somehow associated with the suppression of endoplasmic reticulum stress caused by an APAP overdose.

### 7.2. 3-Phenylpropionic Acid (PPA)

Gut bacteria convert phenylalanine to trans-cinnamic acid, which is subsequently converted to PPA. PPA (0.4% in drinking water) treatment for 4 weeks, followed by a sub-lethal dose of APAP (300 mg/kg, i.p.), could reduce susceptibility toward APAP hepatotoxicity in mice [50]. PPA was shown to suppress the hepatic CYP2E1 protein that catalyzes APAP metabolism to form its toxic metabolite NAPQI. However, the expression of hepatic CYP2E1 genes was similar in the control and PPA-treated animals, implying that PPA regulates CYP2E1 expression post-transcriptionally. Further, PPA (up to 1 mM) did not inhibit the catalytic activity of CYP2E1 in mouse liver microsomes, suggesting PPA itself acts as a substrate of the enzyme and, thus, causes a competitive inhibition of CYP2E1.

### 7.3. Urolithin A

Urolithins are a class of gut microbial metabolites of dietary ellagitannins that exhibit potential antioxidant effects [113]. Amongst them, urolithin A is most important for its potential biological effects [114]. Urolithin A (50, 100, 150, and 300 mg/kg, i.p.) treatment can attenuate acute hepatotoxicity caused by a single APAP (500 mg/kg, i.p.) overdose, evidenced by the significant reduction in the level of AST and ALT in the sera, redox insult in the liver, and hepatocellular necrosis in mice [114]. In search of a protective mechanism, urolithin A was found to endorse mitophagy and activate Nrf2 signaling in the liver. Urolithin A was found to suppress APAP-induced Drp1 elevation and promoted activation of mitophagy proteins, such as Parkin and optineurin expressions in liver. Nrf-2 activation via nuclear translocation can trigger the activation of its downstream antioxidant genes, such as HO-1 and NQO1. The protective role of urolithin A was not affected by mitophagy inhibition, whereas silencing the Nrf-2 gene suppressed its protective role. Thus, it could be concluded that the hepatoprotective role of urolithin A against APAP-induced oxidative stress and hepatocellular necrosis is principally mediated through Nrf-2 activation.

### 7.4. E. lactis IITRHR1 and L. acidophilus Lysates

Pretreatment with *E. lactis* IITRHR1 and *L. acidophilus* lysates prevented APAP-induced cell death in isolated rat hepatocytes [22]. Both the lysates attenuated APAP-induced oxidative stress, evidenced by the reduction in ROS production, nitric oxide level, and lipid peroxidation and enhanced GSH and SOD levels in isolated murine hepatocytes. These postbiotic lysates prevented Bax translocation to mitochondria, Bcl-2 suppression, mitochondrial membrane permeabilization, cytosolic cytochrome C release, caspase 3 activation, DNA fragmentation, and chromatin condensation induced by APAP. In this study, APAP stress-induced hepatocyte death undergoes both apoptosis and necrosis. Pre-treatment with both the bacterial lysates was capable of attenuating both apoptosis and necrosis. Pre- and co-treatment with *E. lactis* IITRHR1 could be able to counteract apoptosis but not necrosis. *L. acidophilus* co-treatment was not found to be effective as pretreatment.

### 7.5. L. fermentum BGHV110 Postbiotic

A postbiotic (HV110) derived from the *L. fermentum* BGHV110 strain could effectively attenuate APAP-induced hepatotoxicity [21]. HV110 (3 mg/mL) co-treatment for 16 h protected HepG2 cells from APAP (50 mM)-induced cell death in both MTT and LDH assays. HV110 co-treatment induced autophagy in HepG2 cells, evidenced by the significant rise in the LC3-II/LC3-I ratio and p62/SQSTM1 degradation (statistically insignificant). The induction of autophagy was shown to prevent APAP-induced hepatocyte damage. HV110 co-treatment with APAP significantly activated mRNA transcriptions of p62/SQSTM1 and PINK1 in HepG2 cells. Thus, this study suggested that HV110 co-treatment prevented APAP-induced hepatotoxicity by increasing PINK1-dependent autophagy in HepG2 cells.

### 7.6. Intracellular Fraction of S. thermophilus TISTR 458

The intracellular fraction of *S. thermophilus* TISTR 458 exhibited APAP-induced hepatocellular injury to HepG2 cells [115]. Additionally, the bacterial intracellular fraction significantly attenuated APAP-induced redox insult to HepG2 cells by endorsing oxygen radical absorbance capacity and endogenous antioxidant levels, such as SOD and GSH. The intracellular fraction of *S. thermophilus* TISTR 458, prepared after incubating the bacteria with 1% prebiotics, such as inulin or fructooligosaccharide, could improve therapeutic efficacy.

To show the hepatoprotective efficacy of the aforementioned postbiotics against APAP hepatotoxicity at a glance, a summary table (Table 3) has been presented.

## 8. Future Scopes

In recent years, research on probiotics and postbiotics has reached new heights. Their health-promoting, disease prevention, and disease curing qualities keep them under the constant focus of nutritionists and physicians. There has been a surge of attention to the use of probiotic and postbiotic supplements as health and disease negotiators. Thus, the market for probiotic and postbiotic supplements is continually growing worldwide. The ability of probiotics to prevent, or treat diseases is always attributed to their local probiotic effects in the gut, which include restoring the integrity of the gut barrier, eliminating pathogenic strains from the body, increasing food digestion and absorption, and reducing irritable bowel syndrome. In addition, they also represent antioxidant, anti-inflammatory, and immunomodulatory effects that participate in disease management. As a result, their disease management properties serve as an extra plus. Postbiotics have a similar mechanism but with minimized risk as compared to probiotics. Since many of these probiotic bacteria and postbiotics are already used as nutraceuticals, their safety profile is not likely to be under question. APAP hepatotoxicity is a common cause of acute liver illness around the world and the lack of suitable treatment makes it a serious issue. From different preclinical studies, it is quite obvious that probiotics and postbiotics can serve as clinically effective hepatoprotective agents. useful against different liver diseases, including APAP hepatotoxicity. However, there are several lacunae in these reports. Although the preclinical studies on the protective roles of probiotics and postbiotics against APAP hepatotoxicity were conducted in 2011, their protective mechanisms were not adequately addressed. Thus, adequate research is required to reveal their protective mechanism, followed by clinical trials to develop them into drug candidates to treat APAP hepatotoxicity. Moreover, there is scope to develop a safe therapeutic approach against APAP hepatotoxicity using these functional foods as therapeutic negotiators, which will open a new avenue in the pharmaceutical/nutraceuticals industry.

## 9. Conclusions

**Figure 4:**
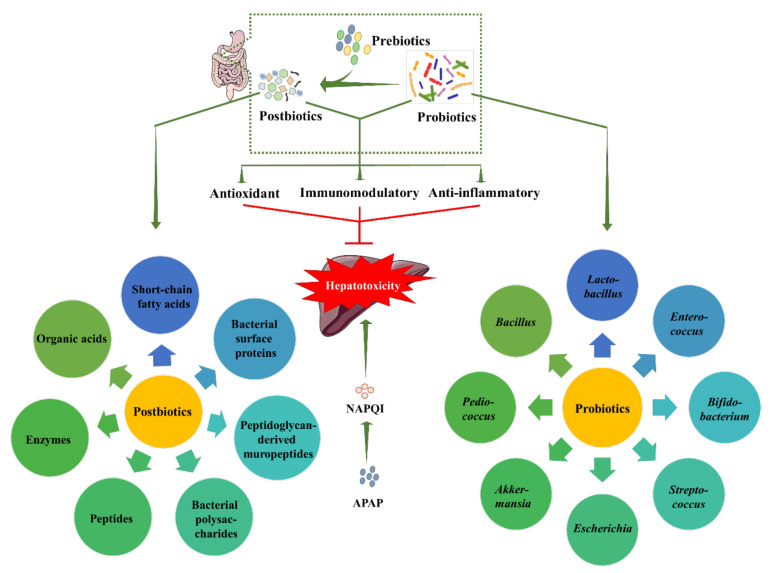
A schematic view of overall protective roles of probiotics and postbiotics against APAP hepatotoxicity. Beneficial bacterial species, known as probiotics, use their innate probiotic capabilities to improve liver health. Some species under the genus *Lactobacillus*, *Bifidobacterium*, *Bacillus*, *Streptococcus*, *Enterococcus*, *Akkermansia*, *Escherichia*, *Pediococcus*, etc., have been categorized as probiotic bacteria. Postbiotics are either microbial components or soluble biologically active metabolites that are generally produced by probiotics by using prebiotics. Probiotics and postbiotics ensure hepatoprotective effect against APAP-induced hepatotoxicity by the antioxidant, anti-inflammatory, and immunomodulatory mechanisms. Green arrows represent downstream events. APAP: acetaminophen, NAPQI: N-acetyl p-benzoquinone imine.

APAP, due to its safety profile, is the most common prescription medicine around the world for the treatment of pain and fever. However, a single overdose, or frequent application of APAP, can cause serious liver injury. Despite the fact APAP hepatotoxicity has long been recognized, it is the drug of choice for physicians, even for children. Thus, it is important to find an effective therapeutic way to reduce or combat APAP hepatotoxicity. N-acetylcysteine is the only approved drug clinically used against APAP hepatotoxicity. However, it represents a very narrow therapeutic window. Therefore, there is an urgent need to find a potential therapeutic strategy against APAP hepatotoxicity. Considering both nutritive and therapeutic benefits, probiotics are categorized as nutraceuticals. Nutraceuticals are gaining popularity around the world for improving physical health and disease management. The hepatoprotective role of different probiotics against different liver diseases, including APAP hepatotoxicity, has been revealed. In addition to their inherent mechanisms, such as reconditioning gut microbiota, maintaining gut barrier rigidity, and preventing bacterial translocation to the liver, probiotics also exhibit antioxidant, immunomodulatory, and anti-inflammatory mechanisms to counteract APAP hepatotoxicity. In addition, some bacteria present in the probiotic-mediated improved gut microbiota produce some beneficial bacterial metabolites that can regulate different signaling events during the process of hepatoprotection. Recently, the term postbiotics, which refers to portions of probiotics, or their metabolic byproducts, emerged, due to their potential health benefits. Postbiotics are gaining popularity as nutraceuticals over probiotics due to their defined chemical identity, safety profile being better than that of living bacteria, and long shelf-life. The hepatoprotective role of postbiotics against APAP hepatotoxicity has been revealed in different preclinical studies. Different postbiotics exhibit different hepatoprotective mechanisms against APAP hepatotoxicity. In this review, we have discussed the hepatoprotective effect of different probiotics and postbiotics against APAP hepatotoxicity, along with their mechanisms of action. Figure 4 depicts a schematic overview of the protective roles of the probiotics and postbiotics against APAP hepatotoxicity. Since both probiotics and postbiotics are popular nutraceuticals around the world, and are typically regarded as safe and well-tolerated, the dietary supplement of probiotics/postbiotics could serve as a novel therapeutic option against APAP toxicity. Adequate research and clinical trials on this topic could lead to the development of a potentially beneficial functional food to control APAP toxicity in people.

## Figures and Tables

**Table 1:** A summary table representing how the composition of the gut microbiota differentially affects APAP hepatotoxicity.

Sl No.	Microorganisms	Animals	Observations	References
1.	Gut microbiota	Male BALB/C and BALB/C germ-free mice	Diurnal variation is linked to gut microbiota and has a major impact on APAP hepatotoxicity. Gut microbiota-derived 1-phenyl-1,2-propanedione endorses APAP hepatotoxicity to some extent by depleting GSH levels resulting in augmented oxidative stress and JNK activation. Treatment of *Saccharomyces cerevisiae* attenuates APAP hepatotoxicity by reducing 1-phenyl-1,2-propanedione production.	[70]
2.	Gut microbiota	C57BL/6 mice and germ-free C57BL/6 mice	Germ-free mice exhibit better tolerability in APAP overdose than non-germ-free mice	[76]
3.	Gut microbiota	BALB/C and BALB/C germ-free and specific-pathogen-free mice	Specific-pathogen-free mice are more susceptible to APAP hepatotoxicity than germ-free mice that exhibit lower expressions of CYP-1A2 and CYP-3A4 enzymes.	[69]
4.	Gut microbiota	C3H/HeH and C3H/HeH germ-free mice	Intestinal microbiota does not reveal any significant difference in susceptibility to APAP hepatotoxicity. However, germ-free mice showed lower hepatotoxicity than non-germ-free mice, which may be associated with decreased TLR4/LPS signaling.	[71]
5.	Gut microbiota (dysbiotic gut)	C57BL/6J and dysbiotic Nlrp6 deficient mice	Increase APAP hepatotoxicity in dysbiotic mice compared to wild-type mice.	[65,66]
6.	Gut microbiota with the abundance of *Mucispirillum* sp., *Turicibacter* sp. and *Ruminococcus* sp.	C57BL/6 mice	Increase APAP hepatotoxicity	[67]
7.	Gut microbiota (α-diversity)	Male C57BL/6 mice	Fructose supplement increases the α-diversity of the gut microbiome resulting in suppression of APAP hepatotoxicity. This altered gut microbiota with the abundance of *Anaerostipes* sp. suppresses CYP-1A2 and CYP-3A4 enzymes and activates GSH.	[72]
8.	Gut microbiota with low Firmicutes/Bacteroidetes ratio and high Proteobacteria proportion, as well as the abundance of *Roseburia* sp., *Lactobacillus* sp., and *Akkermansia* sp. and lower Firmicutes/Bacteroidetes ratio	Male C57BL/6 mice	Monoclonal anti-TLR4 antibody treatment altered the composition of gut microbiota. Fecal transplantation microbiota derived from anti-TLR4 antibody-treated mice exhibited better tolerance against acute APAP hepatotoxicity.	[77]

**Table 2:** A summary table representing hepatoprotective roles of probiotics against APAP hepatotoxicity.

Sl No.	Probiotic Strains	Experimental Models	Treatments	Observations	Remarks	References
1.	*E. lactis* IITRHR1	Male Wistar rats	10^9^ CFU/day, p.o. for 7 days followed by APAP (1 g/kg, p.o.) for 14 days.	Blood parameters: AST ↓, ALT ↓, ALP ↓. Liver parameters: hepatocellular necrosis ↓, lipid peroxidation ↓, protein oxidation ↑, reducing potential ↑, SOD ↑, CAT ↑, GPx ↑, GST ↑, GSH/GSSG ↑, Bax ↓, Bcl-2 ↑, cytochrome C ↓, caspase 9 ↓, caspase 3 ↓, DNA fragmentation ↓	The specific mechanism of action was not revealed. Inadequate data to reveal the exact nature of cell death caused by APAP.	[80]
2.	*S. salivarius* ssp *thermophilus* St.sa	Female Wistar rats	10^9^ CFU/day, p.o. for 7 days followed by a single dose of APAP (200 mg/kg, p.o.) on day 7.	Blood parameters: AST ↓, ALT ↓, ALP ↓. Liver parameters: lipid peroxidation ↓, SOD ↑, CAT ↑, GSH ↑.	Preliminary report, the specific mechanism of action was not revealed.	[79]
3.	*Bacillus* spore blend comprising *B. licheniformis*, *B. indicus*, *B. subtilis*, *B. clausii*, and *B. coagulans* spores	Male Charles River Wistar white rats	10^9^ CFU/day, p.o. for 12 days followed by a single dose of APAP (2 g/kg, p.o.) on day 11.	Blood parameters: AST ↓, ALT ↓, TNF-α ↓, IL-1β ↓, ZO-1 ↓, total antioxidant capacity ↑.	Preliminary report, the specific mechanism of action was not revealed.	[78]
4.	*L. ingluviei* ADK10	Male Wistars rats	10^9^ CFU/day, p.o. for 7 days and co-treatment of APAP (500 mg/kg, i.p.) for 7 days.	Blood and liver parameters: lipid peroxidation ↓, SOD ↑, CAT ↑, GSH ↑.	Preliminary report, the specific mechanism of action was not revealed.	[84]
5.	*L. acidophilus* LA14	Male C57BL/6J mice	6 × 10^8^ CFU/day, p.o. followed by a single dose of APAP (300 mg/kg, p.o.) on day 7.	Blood parameters: Total protein ↑, AST ↓, cholinesterase ↓, total bile acids ↓, total bilirubin ↓, IL-1α ↓. Liver parameters: hemorrhage ↓, nuclear shrinkage ↓, inflammatory cell infiltration ↓.	The specific mechanism of hepatoprotective action was revealed in another model resembling APAP hepatotoxicity.	[86]
6.	*L. rhamnosus* GG	C57BL/6 Mice	2 × 10^8^ CFU/day for 14 days followed by a single dose of APAP (300 mg/kg, p.o.) on day 14.	Blood parameters: AST ↓. Liver parameters: hepatocellular necrosis ↓, GSH/GSSG ↑, Nrf-2 ↑, NQO1↑, HO-1 ↑, GCLC ↑.	Metabolic byproduct of bacteria 5-methoxyindoleacetic acid activates Nrf-2 and its downstream antioxidants.	[73]
7.	*A. muciniphila*	Male Specific pathogen-free C57BL/6 mice	3 × 10^9^ CFU/day for 2 weeks followed by a single dose of APAP (300 mg/kg, i.p.) on day 15.	Blood parameters: AST ↓, ALT ↓. Liver parameters: hepatocellular necrosis ↓, GSH/GSSG ↑, SOD ↑, IL-1β ↓ IL-2 ↓, IL-6 ↓, TNF-α ↓, phospho-PI3K ↑, phospho-Akt ↑, phospho-ERK ↓, phospho-JNK ↓, Bax ↓, Bcl-2 ↑, DNA fragmentation ↓	The specific mechanism of action was not revealed. Inadequate data to reveal the exact nature of cell death caused by APAP.	[81]
8.	Laktera nature, a probiotic formulation comprising *L*. *Bulgaricus* DWT1, *L. helveticus* DWT2, *L. lactis* DWT3, and *S. thermophilus* DWT4, 5, 6, 7, 8	Male Wistar rats	800 and 1600 mg/kg, p.o. for 2 weeks followed by a single dose of APAP (1.2 g/kg)	Blood parameters: ALT ↓, AST ↓, ALP ↓, γ-glutamyl transferase ↓. Liver parameters: hepatocellular necrosis ↓	The specific mechanism of action was not revealed.	[81]

‘↑’ represents upregulation/increase and ‘↓’ represents down-regulation/decrease.

**Table 3:** A summary table representing hepatoprotective roles of postbiotics against APAP hepatotoxicity.

Sl No.	Postbiotics	Experimental Models	Treatments	Observations	Remarks	References
1.	4-Phenylbutyric acid	C57BL/6J mice	100 and 200 mg/kg, i.p. 1 h before APAP (400 mg/kg, i.p.) treatment 100 and 200 mg/kg, i.p. 1 or 2 h after APAP (400 mg/kg, i.p.) treatment.	Blood parameters: ALT ↓, ammonia ↓. Liver parameters: hepatocellular necrosis ↓, nitrotyrosine ↓, DNA fragmentation ↓, Xbp1 splicing ↓, phospho-JNK ↓. Blood parameters: ALT ↓, ammonia ↓. Liver parameters: hepatocellular necrosis ↓, nitrotyrosine ↓, DNA fragmentation ↓, Xbp1 splicing unchanged, phospho-JNK unchanged.	The specific mechanism of action was not revealed.	[111,112]
		C57BL/6J mice	120 mg/kg, i.p. 4 doses at an interval of 3 h starting at 0.5 h after APAP (450 mg/kg, i.p.) treatment up to 12 h.	Blood parameters: ALT ↓, AST ↓. Liver parameters: ATF6 cleavage ↓, phospho-JNK ↓, BiP ↓, Xbp1 splicing ↓, CHOP ↓, Bax activation ↓, oxidative stress ↓, hepatocellular necrosis ↓.	The specific mechanism of action was not revealed.	
2.	3-Phenylpropionic acid	Male or female C57BL/6 mice	0.4% in drinking water for 4 weeks followed by a dose of APAP (300 mg/kg, i.p.)	Liver parameter: CYP2E1 ↓.	3-Phenylpropionic acid acts as a substrate of CYP2E1 and inhibits its catalytic activity.	[50]
3.	Urolithin A	Male C57BL/6J mice	50, 100, 150, or 300 mg/kg, i.p. along with APAP (500 mg/kg, i.p.)	Blood parameters: ALT ↓, AST ↓. Liver parameters: hepatocellular necrosis ↓, Nrf-2 ↑, HO-1 ↑, NQO1 ↑, mitophagy ↑, Drp1 ↓, Parkin ↑, optineurin ↑.	The protective mechanism is Nrf-2 activation.	[114]
4.	*E. lactis* IITRHR1 and *L. acidophilus* lysates	Isolated rat hepatocytes	Pre-, co-, and post-treatment of individual lysate to cells exposed to APAP at IC_50_ concentration.	ROS ↓, nitric oxide ↓, lipid peroxidation ↓, GSH ↑, SOD ↑, Bax translocation ↓, Bcl-2 ↑, mitochondrial membrane permeabilization ↓, cytosolic cytochrome C release ↓, caspase 3 activation ↓, DNA fragmentation ↓, chromatin condensation ↓.	The effect of pre-, co-, and post-treatment exhibited variable effects. The nature of cell death is questionable.	[22]
5.	*L. fermentum* BGHV110 postbiotic	HepG2 cells	3 mg/mL co-treatment for 16 h with APAP (50 mM)	Hepatocyte death ↓, autophagy ↑, LC3-II/LC3-I ratio ↑, BECN1 ↑, p62/SQSTM1 degradation ↑, PINK1mRNA↑, p62/SQSTM1 mRNA ↑.	The protective mechanism is the activation of PINK1-dependent autophagy	[21]
6.	Intracellular fraction of *S. thermophilus* TISTR 458	HepG2 cells	Co-treatment with APAP (25 mM)	Hepatocyte death ↓, oxygen radical absorbance capacity ↑, SOD ↑, GSH ↑.	Intracellular fraction prepared after incubating the bacteria with 1% prebiotics (inulin or fructooligosaccharide) improves therapeutic efficacy	[115]

‘↑’ represents upregulation/increase and ‘↓’ represents down-regulation/decrease.

## Data Availability

Not applicable.
